# *Lactobacillus reuteri* Maintains a Functional Mucosal Barrier during DSS Treatment Despite Mucus Layer Dysfunction

**DOI:** 10.1371/journal.pone.0046399

**Published:** 2012-09-27

**Authors:** Johan Dicksved, Olof Schreiber, Ben Willing, Joel Petersson, Sara Rang, Mia Phillipson, Lena Holm, Stefan Roos

**Affiliations:** 1 Department of Microbiology, Uppsala BioCenter, Swedish University of Agricultural Sciences, Uppsala, Sweden; 2 Department of Medical Cell Biology, Uppsala University, Uppsala, Sweden; 3 Michael Smith Laboratories, University of British Columbia, Vancouver, British Columbia, Canada; 4 Applied Nutrition and Food Chemistry, Center for Chemistry and Chemical Engineering, Lund University, Lund, Sweden; Indian Institute of Science, India

## Abstract

Treatment with the probiotic bacterium *Lactobacillus reuteri* has been shown to prevent dextran sodium sulfate (DSS)-induced colitis in rats. This is partly due to reduced P-selectin-dependent leukocyte- and platelet-endothelial cell interactions, however, the mechanism behind this protective effect is still unknown. In the present study a combination of culture dependent and molecular based T-RFLP profiling was used to investigate the influence of *L. reuteri* on the colonic mucosal barrier of DSS treated rats. It was first demonstrated that the two colonic mucus layers of control animals had different bacterial community composition and that fewer bacteria resided in the firmly adherent layer. During DSS induced colitis, the number of bacteria in the inner firmly adherent mucus layer increased and bacterial composition of the two layers no longer differed. In addition, induction of colitis dramatically altered the microbial composition in both firmly and loosely adherent mucus layers. Despite protecting against colitis, treatment with *L. reuteri* did not improve the integrity of the mucus layer or prevent distortion of the mucus microbiota caused by DSS. However, *L. reuteri* decreased the bacterial translocation from the intestine to mesenteric lymph nodes during DSS treatment, which might be an important part of the mechanisms by which *L. reuteri* ameliorates DSS induced colitis.

## Introduction

Inflammatory bowel diseases (IBD), including Crohn's disease (CD) and ulcerative colitis (UC), are multifactorial diseases, dependent on host genetics, environment, immune response and the intestinal microbiota. There is substantial evidence implicating the involvement of the intestinal microbiota in IBD [1], however, the mechanism through which bacteria induce inflammation has been elusive. During non-inflammatory conditions, the intestinal microbiota-host relationship is symbiotic, and the defense mechanisms preventing translocation of intestinal bacteria through the mucosa and concomitant immune activation are most likely tightly controlled. These mechanisms have been shown to include a luminal firmly adherent mucus layer as well as loosely adherent mucus [2], [3], [4], in addition to epithelial tight junctions and epithelial secretion of antibacterial peptides [5], [6]. Indeed, secretion of the firmly adherent mucus has been shown to be stimulated by bacterial products through toll-like receptor signaling, underpinning the symbiotic relationship between host and intestinal microbiota [3]. The mucus layers serves as a barrier towards the vast amount of luminal bacteria residing in colon. A recent study actually has showed that the inner firm layer is devoid of or contain very low numbers of bacteria under non-inflammatory conditions [4].

The intestinal microbiota of IBD patients has been shown to differ from that of healthy controls and abundant data indicates that the microbiota in IBD patients changes in both composition and localization [7], [8], [9], [10], [11], [12], [13], [14], [15]. One theory is that disease is the result of an imbalance between protective and harmful bacteria or “dysbiosis” [16]. A major challenge is to identify whether dysbiosis is a secondary phenomenon of IBD or the cause of inflammation [16], as all of the research examining the intestinal microbiota in IBD patients has been performed after development of the disease.

In an animal model of colitis, Dextran Sulphate Sodium (DSS) is administered in the drinking water to induce a diffuse mucosal inflammation of the colon with similar clinical symptoms as UC. We have, using this model, previously shown that oral pretreatment with a mix of *Lactobacillus reuteri* strains prevented onset of colitis in rats [17]. The probiotic treatment suppressed up-regulation of P-selectin in the colonic endothelium, which decreased leukocyte-endothelial cell interactions and concomitant leukocyte recruitment to tissue [17]. In addition, the inflammatory action of platelets was also attenuated, as decreased platelet-endothelial cell interactions were observed [17]. The mechanisms by which oral administration of probiotic bacteria causes down regulation of endothelial adhesion molecules are still unknown.

Further understanding of the compositions of the bacterial communities residing in the two mucus layers of the colon during health and disease will give information with relevance for epithelial-microbe interactions important in homeostasis or induction of colitis. The current study addresses this by investigating bacterial distribution and composition in mucus during onset of colitis, as well as the impact of oral addition of *L. reuteri* on the mucosal microbiota, bacterial distribution and translocation during colitis. Our data showed that the amount and composition of bacteria clearly differed between the mucus layers in the animals not treated with DSS, with significantly higher loads of bacteria in the outer mucus layer. The DSS treatment induced a radical change in both composition and abundance of bacteria and eradicated the observed differences between mucus layers. Treatment with *L. reuteri* protected against colitis, but it did not counteract the changes of the mucus layer integrity or the mucus microbiota caused by DSS. However, *L. reuteri* reduced the translocation of bacteria from the mucosa to the mesenteric lymph nodes, which might be a central part of the explanation how this bacterium protects against DSS induced colitis.

## Materials and Methods

### Ethics Statement

All animal experiments were approved by the Swedish Laboratory Animal Ethical Committee in Uppsala (animal experiments numbers C349/10 and C287/9) and were conducted in accordance with guidelines of the Swedish National Board for Laboratory Animals.

### Animal handling and study design

Twelve male Sprague-Dawley rats (B&K, Sollentuna) weighing between 190 and 290 g (weight before treatment), were kept under standardized conditions at a temperature of 21–22°C and with 12 h light and 12 h dark cycle. The animals were allowed to acclimatize for 1 week before the experiments started. Rats were divided into 4 groups with 3 rats in each group: control, DSS-treated, *L. reuteri* treated and lastly *L. reuteri*+DSS treated. *L. reuteri*-treated rats were given a cocktail of 10^9^ bacteria in 0.5 ml saline containing an equal amount of four strains of *L. reuteri*. This cocktail was given daily by gavage for 16 days. The rats treated with both *L. reuteri* and DSS were given 5% DSS in the drinking water for the last 9 days of their *L. reuteri* treatment.

### Induction and assessment of colitis

Rats were given 5% (wt/wt) DSS (DSS 37–40 kilodaltons; TdB Consultancy, Uppsala, Sweden) in their drinking water for 9 days. The severity of colitis was assessed daily on the basis of clinical parameters including weight loss, stool consistency and blood content, and presented as Disease Activity Index (DAI), a scoring method described in detail by Cooper and coworkers [18].

### Bacterial suspensions

The bacterial cocktail consisted of the following four strains of *L. reuteri*: two isolated from rat, R2LC (kindly provided from Siv Ahrné, Lund University, Sweden) and JCM 5869 (Japan Collection of Microorganisms), and two from human sources, ATCC PTA 4659 and ATCC 55730 (kindly provided from Biogaia, Stockholm, Sweden). The bacteria were cultivated separately in 200 ml MRS broth (Oxoid, Basingstoke, UK) at 37°C for 20 h. Bacterial cells were washed once with PBS, and suspended in 2 ml freezing solution [0.82 g K_2_HPO_4_, 0.18 g KH_2_PO_4_, 0.59 g sodium citrate, 0.25 g MgSO_4_×7 H_2_O, and 172 ml glycerol (87%) diluted to 1000 ml with ddH_2_O]. The bacterial suspensions were mixed and stored at −70°C until use.

### *In vivo* sampling of the mucus layers

The levels of bacteria in the colonic mucus layers and mesenteric lymph nodes (MLN) were quantified in rats from each treatment group. Rats were anaesthetized with 120 mg kg^−1^ body weight of 5-ethyl-5-(1-methylpropyl)-2-thiobutabarbital sodium (Inactin®, Sigma, St. Louis, MO), given intraperitoneally. The colonic preparation for intravital microscopy and mucus measurements have been extensively described previously [2]. Total mucus thickness was measured with micropipettes connected to a micromanipulator (Leitz, Wetzlar, Germany) with a digimatic indicator (IDC Series 543, Mitutoyo, Tokyo, Japan) observed under a stereo-microscope (Leica MZ12, Leica, Heerbrugg, Switzerland). The luminal surface of the mucus gel was visualized by placing graphite particles (activated charcoal, extra pure, Merck, Darmstadt, Germany) on the gel, and the colonic epithelial cell surface was visible through the microscope. After measurements of total mucus thickness the loosely adherent mucus layer was removed by gentle suction, and the sample was snap-frozen in liquid nitrogen and stored at −70°C. The remaining firmly adherent mucus layer thickness was then measured. The descending colon was slightly moved so that a part of the adjacent area was exposed. The loosely adherent mucus was removed without being measured and the firmly adherent mucus scraped off with a scalpel and snap-frozen in liquid nitrogen and stored at −70°C. This procedure was performed to avoid contamination of the firmly adherent mucus by the micropipette. The total volume of the mucus layers was calculated from measurements of mucus thickness and exposed area. The volumes of mucus were between 1 and 16 µl and the mucus samples were suspended in 1 ml PBS pH 7.4. MLN were excised using sterilized forceps, snap frozen in liquid nitrogen and stored at −70°C. Finally, the anaesthetized rats were euthanized by cervical dislocation.

### Culturing of bacteria

One third of the collected mucus samples were homogenized by pipetting and vortexing, while the MLN were homogenized by passing through a 70 µm cell strainer (BD, Stockholm, Sweden). Serially diluted samples were plated on Wilkins-Chalgren Agar (Merck) and Rogosa plates (Merck), and incubated at 37°C for 48 h in anaerobic atmosphere (Gaspack system, BD, Sparks, MD). Colonies were counted, and for the MLN a total of 90 representative colonies were isolated for identification. The isolated bacteria were identified by PCR amplification of the 16S rRNA genes (see below) followed by sequencing of the first 600 bp according to standard procedures.

### DNA isolation

QiaAmp DNA mini kit (Qiagen, Hilden, Germany; tissue protocol) was used to recover DNA from the mucus samples and the FastDNA Spin kit for Soils (MP Biomedicals, Solon, OH, USA) was used to recover DNA from the MLN. A 700 µl suspension of the firmly or loosely adherent mucus layers and 50 mg of MLN were used for the DNA extractions. The procedure was carried out according to the manufacturer's description after homogenizing the samples with bead beating 2×45 s using MP FastPrep-24 (MP Biomedicals) at setting 5.0. The carbon particles that were used to visualize the mucus adsorbed DNA, but by adding 10 µg tRNA and 100 µg of BSA we were able to block the carbon particles prior to the DNA-extraction to optimize DNA recovery.

### Terminal restriction fragment length polymorphism (T-RFLP)

Bacterial 16S rRNA genes were PCR amplified from each extract (two technical replicates per extract). In the PCR, DNA was amplified using PuReTaq Ready-To-Go beads (GE Healthcare, Uppsala, Sweden) under running conditions that have been described elsewhere (Dicksved et al, 2008).

The restriction enzyme *Hae*III (GE Healthcare) was used to digest the PCR products. Digested fragments were separated using an ABI 3730 capillary sequencer as previously described [8]. Relative peak areas were determined by dividing the area of each individual terminal restriction fragment (TRF) with the total peak area. Values between 30 and 500 bp were analyzed. Peaks with a relative abundance of more than 0.5% were considered in the analysis. Technical replicates were compared and only TRFs present in both replicates were included in the analysis.

### Cloning and sequencing

16S rRNA genes were cloned from selected samples to identify the bacteria corresponding to the TRF sizes of interest based on the T-RFLP data. Extracted DNA from loosely and firmly adherent mucus layers from each treatment and from MLN samples (DSS treatment only) were PCR amplified in duplicate under running conditions that have been described above. The duplicate PCR samples were pooled and gel purified using the Qiagen gel extraction kit (Qiagen). Eleven clone libraries were constructed by inserting PCR products into TOPO TA pCR 4.0 vectors (Invitrogen, Carlsbad, CA), followed by transformation into *Escherichia coli* TOP 10 competent cells (Invitrogen). A total of 64 inserts from each library were PCR amplified using vector primers M13f and M13r (Invitrogen) with the same thermal cycling program as described above for T-RFLP analysis. The PCR products were diluted 50-fold and used in a nested PCR reaction with fluorescently tagged Bact-8F and 926r for T-RFLP analysis of inserted clones (see above). Clone TRF sizes were matched with the T-RFLP data matrix and the clones with TRF sizes of interest were selected for sequence analysis, at a total of 150 sequences. The sequences were aligned against GenBank database entries using standard nucleotide BLAST at NCBI (URL: www.ncbi.nlm.nih.gov) and/or against the Ribosomal Database Project 10 Sequence Match, for classification. Unique sequences were deposited in GenBank at NCBI, under the following accession numbers: GU237034–GU237043 and JF794853–JF794989.

### Statistical analysis

ANOVA followed by Tukey's post hoc test was used to test for treatment effects on DAI and bacterial counts. In cases where no bacteria were detected in the MLN, values were set to the detection limit of the method. T-RFLP data from each sample was normalized and entered into a data matrix that consisted of TRFs as variables and sample as objects. A consensus T-RFLP profile from each biological replicate was constructed by averaging the technical duplicates. Bray Curtis metrics was used for cluster analysis of the TRF data, and Kruskal Wallis tests, to identify TRFs that were significantly correlated to a specific cluster. P-values<0.05 were considered significant. To account for multiple comparisons, the false discovery rate was assessed according to the method of Benjamini and Hochberg [19]. Based on global *P* values for all compared variables a 5% false discovery rate identified 5 false positives out of 44 claimed differences. These are indicated with a † in figures.

## Results

### Despite ameliorated colitis, *L. reuteri* does not counteract damage of the mucus barrier function caused by DSS

**Figure 1:**
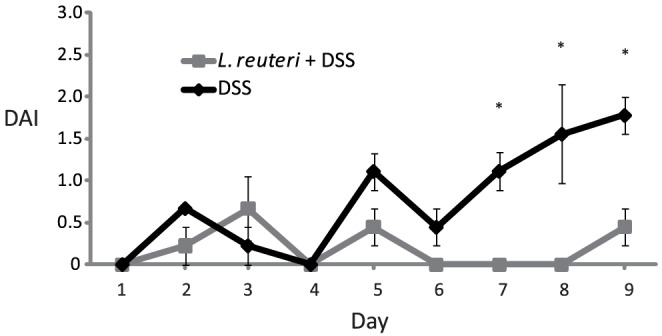
Disease activity index in animals treated with DSS alone or *Lactobacillus reuteri*+DSS. In the first group, DSS were given in the drinking water for 9 days. In the second, 10^9^ *L. reuteri*/day were given by gavage for 16 days and DSS were given for the last 9 days of the treatment. *p<0.05 DSS vs. *L. reuteri*+DSS.

Consistent with our previous study [17] pre-treatment with *L. reuteri* prior to DSS colitis induction reduced DAI (*P*<0.05) the last 3 days of colitis induction compared to animals receiving only DSS (Figure 1). Animals in the control group and *L. reuteri* group had a DAI of 0 for all 9 days.

**Figure 2:**
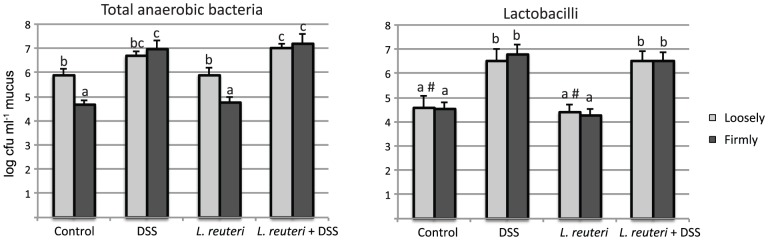
Numbers of total anaerobic bacteria and lactobacilli determined by cultivation. For each treatment group (controls, DSS, *L. reuteri*, *L. reuteri*+DSS), data are shown for both loosely and firmly adherent colonic mucus. Different letters (a–c) above the bars indicate significant differences in bacterial counts between groups or mucus layers (p<0.05). # Lactobacilli were only detected in two of the three samples.

By culturing samples of the different colonic mucus layers under anaerobic conditions, we found that the total number of bacteria was significantly higher in the loosely adherent layer than in the firmly adherent layer in untreated and *L. reuteri* treated rats (Figure 2). Interestingly, administration of DSS abolished the barrier function of the firmly adherent mucus, as upon DSS treatment it was colonized to a similar extent as the loosely adherent mucus. Consequently bacterial counts were higher in the firmly adherent mucus of DSS treated compared to controls and *L. reuteri* treated rats (Figure 2). However, this was also observed in the DSS treated rats pretreated with *L. reuteri,* which developed a very mild colitis. In addition, the number of lactobacilli increased significantly in both the loosely and firmly adherent mucus following DSS-treatment but irrespective of *L. reuteri* pretreatment, suggesting that DSS favors the growth or colonization of lactobacilli. The *L. reuteri* treatment, however, did not increase the number of lactobacilli (Figure 2).

### DSS alters the colonic microbiota and obliterates the microbial differences between mucus layers

**Figure 3:**
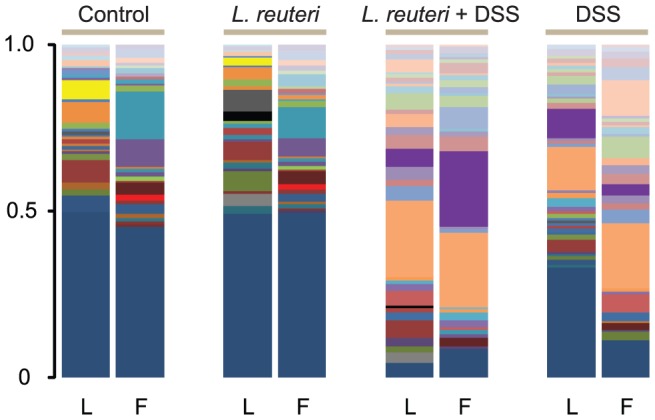
Profiling of the microbial structure in colonic mucus layers by T-RFLP. The columns show representative profiles from the loose (L) and firm (F) colonic mucus layers and treatment groups (control, *L. reuteri, L. reuteri*+DSS and DSS).

**Figure 4:**
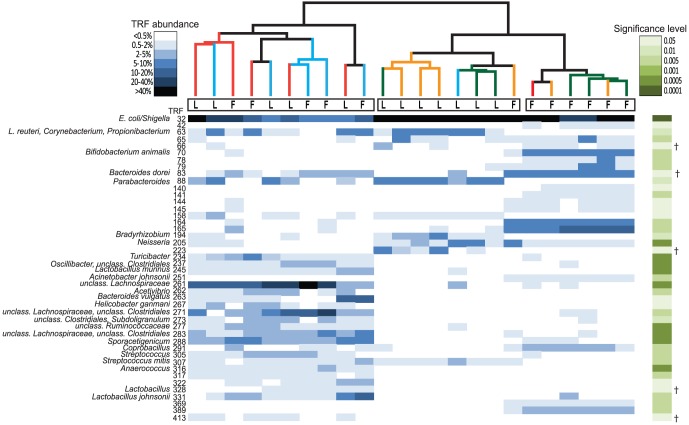
Clustering of samples according to its microbial structure. The heatmap shows the relative abundances of TRFs that differed significantly between clusters. The base pair (bp) sizes are indicated in the figure. Each column represents the abundance profile for a sample and each row the abundance profile for a TRF. The abundance interval is shown in a blue color scale and significance levels in a green color scale. †Indicates TRFs that were identified in false discovery rate analysis. Samples were sorted according to clustering using Bray Curtis metrics. Branch color represents: red, DSS; blue, *L. reuteri*+DSS; green, control; and yellow, *L. reuteri*. T-RFLP data of loosely adherent mucus (L) and firmly adherent mucus (F) showing one cluster of control and *L. reuteri* firm mucus samples (right cluster), one cluster of control and *L. reuteri* loose mucus samples (middle cluster) and one cluster of DSS-treated animals including a mix of both loosely and firmly mucus (left cluster).

The T-RFLP data revealed that the bacterial composition in the loosely adherent mucus in control and *L. reuteri* treated groups was distinct from the firmly adherent mucus (Figure 3). Cluster analysis of the T-RFLP data yielded three discrete clusters (Figure 4). Samples from loosely adherent mucus from control and *L. reuteri* treated groups appeared as one cluster, while samples from firmly adherent mucus from the same two groups appeared as another cluster (Figure 4). Thus, the two mucus layers differed not only in the number of bacteria they harbor, but also in terms of which bacteria they harbor. *L. reuteri* treatment did not change the microbiota compared to non-treated controls, indicating that the protective effect of *L. reuteri* was not linked to a substantial change of the dominant microbial members. Interestingly, not only did samples from the two groups receiving DSS cluster together, they did so with no discrimination between the loosely- and firmly adherent mucus layers (Figures 3 and 4). This is consistent with the culturing data from the mucus layers, which showed that DSS destroys the barrier function of the firmly adherent mucus, making it permissive to colonization at a similar extent as the loosely adherent mucus. The T-RFLP data shows that the difference in microbial composition between the two mucus layers was abolished when DSS was given.

### Microbial signatures discriminate between clusters

We were further interested in identifying bacterial species that were linked to either the DSS treatment or the different mucus layers. Clone libraries were created to obtain sequence data for correlation between TRFs and corresponding bacterial identities. In total 704 clones were screened for their respective TRF size and only the clones with a TRF size of interest were sequenced. Several TRFs were significantly correlated to either the firm, loose or DSS cluster. Sequence data of clones with matching TRF sizes are shown in Table 1. The DSS treatment was associated with the most pronounced shift in the microbiota with significantly higher abundance of several TRFs, primarily belonging to the *Firmicutes* phylum. These sequences were mostly classified in the orders *Clostridiales* and *Lactobacillales*. In addition, TRFs matched with *Helicobacter ganmani* and *Bacteroides vulgatus* were positively correlated with the DSS treatment. The TRFs identified as *Proteobacteria,* of which the majority of sequences matched with *E. coli*/*Shigella,* were negatively correlated with the DSS treatment.

**Table 1:** Correlation of individual TRFs with their corresponding bacteria to different correlation clusters.

Cluster	TRF (bp)	Best sequence identity (Phylum)	Correlation	P-value
**DSS cluster**	32	*Escherichia coli*/*Shigella* **(P)**	**−**	0.00001
	205	*Neisseria* spp. **(P)**	**−**	0.00013
	234	*Turicibacter* spp. **(F)**	**+**	0.0002
	237	*Oscillibacter*, unclassified *Clostridiales* **(F,F)**	**+**	0.0002
	245	*Lactobacillus murinus* **(F)**	**+**	0.0002
	261	unclassified *Lachnospiraceae* **(F)**	**+**	0.0002
	262	*Acetivibrio* spp. **(F)**	**+**	0.004
	263	*Bacteroides vulgatus* **(B)**	**+**	0.016
	267	*Helicobacter ganmani* **(P)**	**+**	0.016
	271	unclassified *Clostridiales* **(F)**	**+**	0.001
	273	unclassified *Clostridiales*, *Subdoligranulum* **(F,F)**	**+**	0.005
	277	unclassified *Ruminococcaceae* **(F)**	**+**	0.0002
	283	unclassified *Lachnospiraceae*, unclassified *Clostridiales* **(F,F)**	**+**	0.0003
	288	*Sporacetigenicum* spp. **(F)**	**+**	0.0001
	305	*Streptococcus* spp. **(F)**	**+**	0.0012
	316	*Anaerococcus* spp. **(F)**	**+**	0.0002
	331	*Lactobacillus johnsonii* **(F)**	**+**	0.001
				
**Firm mucus**	63	*L. reuteri*, *Propionibacterium*, *Corynebacterium* **(F,A,A)**	**−**	0.0096
**cluster**	70	*Bifidobacterium animalis* **(A)**	**+**	0.0015
	251	*Acinetobacter johnsonii* **(P)**	**+**	0.001
	271	*Coprobacillus* spp. **(F)**	**+**	0.001
	307	*Streptococcus mitis* **(F)**	**−**	0.0022
				
**Loose mucus**	88	*Parabacteroides* spp. **(B)**	**+**	0.009
**cluster**	194	*Bradyrhizobium* spp. **(P)**	**+**	0.006

Samples in the firm mucus cluster of non-DSS treated rats were associated with higher abundances of *Bifidobacterium animalis*, *Acinetobacter johnsonii* and *Coprobacillus* spp. compared with the loose mucus and DSS cluster. Conversely, lower abundances of *Lactobacillus reuteri*, *Corynebacterium* spp, *Propionibacterium* spp. and *Streptococcus mitis* were associated with the firm mucus cluster. The loose mucus cluster was associated with higher abundances of TRFs identified as *Parabacteroides* spp. and *Bradyrhizobium*.

### *L. reuteri* reduces DSS associated bacterial translocation to mesenteric lymph nodes

**Figure 5:**
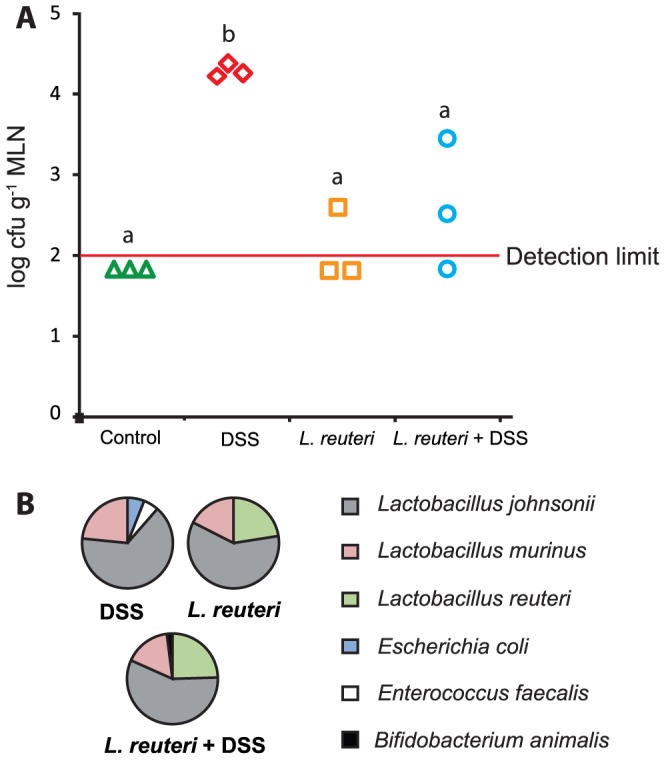
*L. reuteri* reduces DSS associated translocation. A) Culture data (log cfu g^−1^ mesenteric lymph node) from mesenteric lymph nodes from the different treatment groups. B) The identity of the dominant culturable bacteria that translocated. Different letters (a–b) above the bars indicate significant differences in bacterial counts between groups (p<0.05). ANOVA followed by Tukey's post hoc test was used for the statistical analysis.

Bacterial culturing of the MLN samples from the control group resulted in levels below the detection limit of 100 cfu g^−1^ MLN (Figure 5A). In samples from the *L. reuteri* group, low numbers of bacteria were detected in only one out of three samples (Figure 5A). However, all three samples from the DSS group contained significantly higher levels of bacteria than all other groups, indicating a high level of bacterial translocation. This effect was significantly reduced by *L. reuteri* pretreatment where only two out of three samples contained detectable amounts of bacteria (Figure 5A). *L. reuteri* thus decreases translocation of bacteria in DSS-induced colitis. Sequencing of the 16S rRNA genes of the individual colonies found in the three groups showed that the dominating cultivable bacterium was *Lactobacillus johnsonii,* which accounted for more than 60% in each of the three groups where colonies were found (Figure 5B). Another *Lactobacillus* sp., *Lactobacillus murinus* (or possibly *Lactobacillus animalis*) accounted for approximately 20% in each of the three groups. In the DSS group, the remaining bacteria were *Escherichia/Shigella* sp. and *Enterococcus faecalis*, each accounting for approximately 5% of cultured bacteria. In the two groups receiving probiotic treatment, *L. reuteri* accounted for approximately 25% of translocated cultivable bacteria.

**Figure 6:**
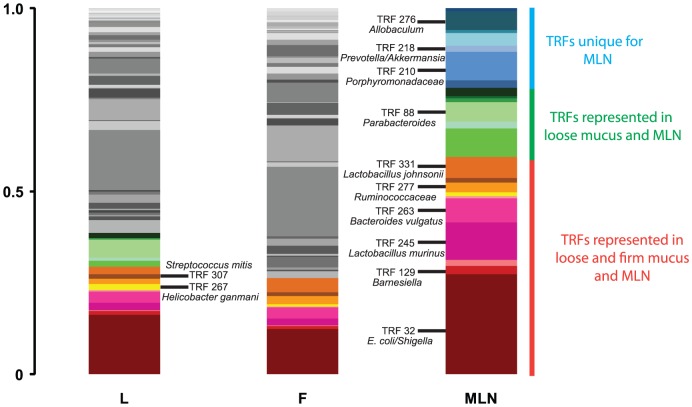
Consensus T-RFLP profiles from different sampling sites in the DSS treated animals. TRFs colored in a red color scale were found in loose (L), firm (F) mucus and the mesenteric lymph nodes (MLN), TRFs colored in a green color scale were found in loose mucus and MLN, TRFs colored in a blue scale were unique for the MLN and TRFs colored in grey were only found in mucus.

Due to a low number of bacteria in the MLN, reproducible T-RFLP profiles could only be recovered from the three animals treated with DSS. Many TRFs were detected in both loose and firm mucus as well as the MLN from the same animal and these TRFs represented over 50% of the total TRF abundance in the lymph node samples (Figure 6). Foremost, the bacteria that were detected at all sites were *E. coli/Shigella,* numerous lactobacilli, *Helicobacter ganmani*, *Streptococcus mitis, Bacteroides vulgatus, Barnesiella* and *Ruminococcaceae*. Interestingly, some of the TRFs were unique for the lymph node samples (Figure 6). They were identified as *Allobaculum*, *Porphyromonadaceae*, *Prevotella* and *Akkermansia.*

## Discussion

For the first time, this study established the difference not only in the number of bacteria residing in the colonic firmly adherent mucus versus the loosely adherent mucus, but also in the composition of the microbiota of the two layers. The constitution of the bacterial communities differed in the two colonic mucus layers, and fewer bacteria were found in the firmly adherent mucus. However, induction of colitis eradicated the differences in bacterial number as well as composition between the two mucus layers. A similar effect was seen in animals that had been treated with *L. reuteri* prior to the DSS treatment. However, these animals did not develop colitis, indicating that microbial changes were not a product of colitis, which has previously been reported [20]. This also indicated that the protective effect of *L. reuteri* in DSS induced colitis was not associated with improvement of the mucus barrier.

It has previously been suggested that the inner firmly adherent colonic mucus layer acts as a barrier towards luminal bacteria and is devoid of bacteria [4]. Our results partially support that data, since we show that the bacterial concentration of the firmly adherent mucus is approximately 1/10 of the concentration in the loosely adherent mucus in healthy rats. Yet, we detected bacteria in the firmly adherent mucus layer using both culture based and culture independent methods. The differences between these results and those of Johansson and coworkers [4] could be explained by the fact that different detection techniques were used. One consideration of our protocol was the risk of cross contamination when collecting the individual mucus layers. Yet, this is not likely since the T-RFLP data from the two layers showed different bacterial profiles and indicates that only certain bacteria are able to penetrate or colonize the firmly adherent mucus. Although we did not study the spatial organization of the bacteria in the inner firm mucus layer, it is certainly plausible that the bacteria may have been on the outer portion and not evenly distributed within the inner firm mucus. Another explanation might be that the FISH method used by Johansson and coworkers did not detect all bacteria, which is supported by their analysis of firmly adherent mucus with semi-quantitative PCR where an amplification with 30 PCR cycles showed the presence of bacteria.

Addition of *L. reuteri* alone did not influence the microbial community composition or decrease the abundance of potential deleterious bacteria. Neither did *L. reuteri* influence the total number of lactobacilli in the mucus (Figure 2). Thus, lactobacilli already colonizing the colonic mucus did not have the same protective effect as the added cocktail, indicating that the effect was attributed to the presence of certain *Lactobacillus* strains and not a general increase of the number of lactobacilli.

It has previously been reported that dysbiosis occurs in experimental colitis [20], [21]. The present study demonstrates that DSS induced colitis caused dysbiosis and obliterated the difference in abundance and structure of the microbiota between mucus layers. Several TRFs were significantly correlated to the DSS treatment. Most striking was that the relative abundance of TRF 32, identified as *E. coli/Shigella*, was significantly lower in the DSS treated animals. One must keep in mind that the total abundance of bacteria in the mucus layer of the DSS treated animals was approximately 10–100 times higher compared to the control group (Figure 2), thus DSS treated rats had higher total counts of *E. coli/Shigella*. Several studies have also reported increased abundance of *E. coli* or *Enterobacteriaceae* in intestinal inflammation [7], [11], [12], [20], [22]. Further, an increased abundance of members of the *Firmicutes* phylum was strongly associated with the DSS cluster (Table 1). Many of these bacteria have not previously been correlated with colitis or inflammation. One report, however, recently identified the expansion of an unclassified *Lachnospiraceae,* highly similar to the mucin degrading bacteria *Ruminococcus gnavus,* in biopsy samples of Crohn's patients [11]. In addition, flagellated members of the *Lachnospiraceae* family has previously been highlighted in experimental colitis [23]. Interestingly, three of the TRFs that were correlated with the DSS cluster were identified as unclassified *Lachnospiraceae* phylotypes (TRF 261, TRF 271 and TRF 283). However, it is possible that the observed changes in the microbiota seen after DSS treatment are independent of the inflammation. Animals treated with DSS alone or in combination with *L. reuteri* clustered together (Figure 4) despite a difference in inflammatory status. One possible explanation of the dysbiosis seen in our DSS model is that some members of the gut microbiota could be favored by utilizing DSS as a substrate.

If the colonic mucosa displays severe ulcerations during colitis, bacteria may translocate directly into the circulation, but since the rat DSS model induces only relatively mild epithelial damage, MLN are well suited sample points to assess the level of bacterial translocation [24]. Interestingly, treatment with *L. reuteri* significantly reduced bacterial translocation in the DSS model. Since no significant changes in microbial composition or mucus barrier function were seen with supplement of *L. reuteri*, the protection appears to be situated in the epithelial barrier. The probiotic mix VSL#3 has previously been shown to ameliorate colitis by maintaining tight junction protein expression [25]. Another possible mechanism for the strengthening of the barrier could be associated with an altered expression of membrane-bound mucins. Probiotic lactobacilli have been shown to adhere to intestinal epithelial cells in the duodenum and induce transcription of membrane bound mucin MUC3 [26]. Another *in vitro* study showed that probiotics inhibit *E. coli* adherence by inducing mucin gene expression [27].

The composition of translocated bacteria was characterized both with identification of isolated strains by 16S rRNA gene sequencing and by T-RFLP analysis of MLN. Lactobacilli, and in particular, *L. johnsonii*, were the dominant fraction among the isolated bacteria, regardless of treatment (Figure 5B). In the T-RFLP analysis, the TRF corresponding to *E. coli/Shigella* was dominant, but TRFs corresponding to some of the lactobacilli detected by culturing were also predominant. The majority of the TRFs in the MLN from the DSS treated animals were less abundant in the mucus samples, indicating that some bacteria in the mucus are more capable than others to migrate across the epithelial layer. The number of bacterial species detected with the T-RFLP analysis was considerably higher than the number detected with cultivation. However, cultivation enabled a quantification of the bacteria and also the detection of *L. reuteri* as one of the translocated bacteria. Together these two methods provide a good overview of the identities of translocated bacteria.

We show in this study that the firmly adherent mucus holds an important and selective barrier function, protecting the epithelium from the vast number of bacteria residing in the gut. DSS destroys this mucus barrier, eradicating the differences in bacterial number as well as composition between the two mucus layers. The colonic epithelium is subsequently exposed to a vast array of gut bacteria that are not normally in contact with the epithelium. The addition of *L. reuteri* prevents DSS-colitis in rats, but the protective effect is not linked to a strengthened mucus barrier or a counterbalance of dysbiosis, but is instead attributed to a functional strengthening of the epithelial barrier, thus reducing bacterial translocation to the MLN.

## References

[1] Packey CD, Sartor RB (2009) Commensal bacteria, traditional and opportunistic pathogens, dysbiosis and bacterial killing in inflammatory bowel diseases. Curr Opin Infect Dis 22: 292–301. 19352175 10.1097/QCO.0b013e32832a8a5d PMC2763597

[2] Atuma C, Strugala V, Allen A, Holm L (2001) The adherent gastrointestinal mucus gel layer: thickness and physical state in vivo. Am J Physiol Gastrointest Liver Physiol 280: G922–929. 11292601 10.1152/ajpgi.2001.280.5.G922

[3] Petersson J, Schreiber O, Hansson GC, Gendler SJ, Velcich A, et al (2011) Importance and regulation of the colonic mucus barrier in a mouse model of colitis. Am J Physiol Gastrointest Liver Physiol 300: G327–333. 21109593 10.1152/ajpgi.00422.2010 PMC3302190

[4] Johansson ME, Phillipson M, Petersson J, Velcich A, Holm L, et al (2008) The inner of the two Muc2 mucin-dependent mucus layers in colon is devoid of bacteria. Proc Natl Acad Sci U S A 105: 15064–15069. 18806221 10.1073/pnas.0803124105 PMC2567493

[5] Jager S, Stange EF, Wehkamp J (2010) Antimicrobial peptides in gastrointestinal inflammation. Int J Inflam 2010: 910283. 21151692 10.4061/2010/910283 PMC2992817

[6] Marchiando AM, Graham WV, Turner JR (2010) Epithelial barriers in homeostasis and disease. Annu Rev Pathol 5: 119–144. 20078218 10.1146/annurev.pathol.4.110807.092135

[7] Baumgart M, Dogan B, Rishniw M, Weitzman G, Bosworth B, et al (2007) Culture independent analysis of ileal mucosa reveals a selective increase in invasive *Escherichia coli* of novel phylogeny relative to depletion of *Clostridiales* in Crohn's disease involving the ileum. ISME journal 1: 403–418. 18043660 10.1038/ismej.2007.52

[8] Dicksved J, Halfvarson J, Rosenquist M, Järnerot G, Tysk C, et al (2008) Molecular analysis of the gut microbiota of identical twins with Crohn's disease. ISME J 2: 716–727. 18401439 10.1038/ismej.2008.37

[9] Gophna U, Sommerfeld K, Gophna S, Doolittle WF, Veldhuyzen van Zanten SJ (2006) Differences between tissue-associated intestinal microfloras of patients with Crohn's disease and ulcerative colitis. J Clin Microbiol 44: 4136–4141. 16988016 10.1128/JCM.01004-06 PMC1698347

[10] Seksik P, Rigottier-Gois L, Gramet G, Sutren M, Pochart P, et al (2003) Alterations of the dominant faecal bacterial groups in patients with Crohn's disease of the colon. Gut 52: 237–242. 12524406 10.1136/gut.52.2.237 PMC1774977

[11] Willing B, Dicksved J, Halfvarson J, Andersson A, Lucio M, et al (2010) A pyrosequencing study in twins shows that gastrointestinal microbial profiles vary with inflammatory bowel disease phenotypes. Gastroenterology 139: 1844–1854. 20816835 10.1053/j.gastro.2010.08.049

[12] Willing B, Halfvarson J, Dicksved J, Rosenquist M, Järnerot G, et al (2009) Twin studies reveal specific imbalances in the mucosa-associated microbiota of patients with ileal Crohn's disease. Inflamm Bowel Dis 15: 653–660. 19023901 10.1002/ibd.20783

[13] Frank DN, St Amand AL, Feldman RA, Boedeker EC, Harpaz N, et al (2007) Molecular-phylogenetic characterization of microbial community imbalances in human inflammatory bowel diseases. Proc Natl Acad Sci U S A 104: 13780–13785. 17699621 10.1073/pnas.0706625104 PMC1959459

[14] Manichanh C, Rigottier-Gois L, Bonnaud E, Gloux K, Pelletier E, et al (2006) Reduced diversity of faecal microbiota in Crohn's disease revealed by a metagenomic approach. Gut 55: 205–211. 16188921 10.1136/gut.2005.073817 PMC1856500

[15] Qin J, Li R, Raes J, Arumugam M, Burgdorf KS, et al (2010) A human gut microbial gene catalogue established by metagenomic sequencing. Nature 464: 59–65. 20203603 10.1038/nature08821 PMC3779803

[16] Tamboli CP, Neut C, Desreumaux P, Colombel JF (2004) Dysbiosis in inflammatory bowel disease. Gut 53: 1–4. 14684564 10.1136/gut.53.1.1 PMC1773911

[17] Schreiber O, Petersson J, Phillipson M, Perry M, Roos S, et al (2009) *Lactobacillus reuteri* prevents colitis by reducing P-selectin-associated leukocyte- and platelet-endothelial cell interactions. Am J Physiol Gastrointest Liver Physiol 296: G534–542. 19147805 10.1152/ajpgi.90470.2008

[18] Cooper HS, Murthy SN, Shah RS, Sedergran DJ (1993) Clinicopathologic study of dextran sulfate sodium experimental murine colitis. Lab Invest 69: 238–249. 8350599

[19] Benjamini Y, Hochberg Y (1995) Controling the false discovery rate: a practical and powerful approach to multiple testing. JR Stat Soc Series B Methodol 57: 289–300.

[20] Lupp C, Robertson ML, Wickham ME, Sekirov I, Champion OL, et al (2007) Host-mediated inflammation disrupts the intestinal microbiota and promotes the overgrowth of *Enterobacteriaceae* . Cell Host Microbe 2: 204. 18030708 10.1016/j.chom.2007.08.002

[21] Lamine F, Eutamene H, Fioramonti J, Bueno L, Theodorou V (2004) Colonic responses to *Lactobacillus farciminis* treatment in trinitrobenzene sulphonic acid-induced colitis in rats. Scand J Gastroenterol 39: 1250–1258. 15743003 10.1080/00365520410007953

[22] Darfeuille-Michaud A, Neut C, Barnich N, Lederman E, Di Martino P, et al (1998) Presence of adherent *Escherichia coli* strains in ileal mucosa of patients with Crohn's disease. Gastroenterology 115: 1405–1413. 9834268 10.1016/s0016-5085(98)70019-8

[23] Ye J, Lee JW, Presley LL, Bent E, Wei B, et al (2008) Bacteria and bacterial rRNA genes associated with the development of colitis in IL-10(−/−) mice. Inflamm Bowel Dis 14: 1041–1050. 18381614 10.1002/ibd.20442 PMC3804113

[24] McGuckin MA, Eri R, Simms LA, Florin TH, Radford-Smith G (2009) Intestinal barrier dysfunction in inflammatory bowel diseases. Inflamm Bowel Dis 15: 100–113. 18623167 10.1002/ibd.20539

[25] Mennigen R, Nolte K, Rijcken E, Utech M, Loeffler B, et al (2009) Probiotic mixture VSL#3 protects the epithelial barrier by maintaining tight junction protein expression and preventing apoptosis in a murine model of colitis. Am J Physiol Gastrointest Liver Physiol 296: G1140–1149. 19221015 10.1152/ajpgi.90534.2008

[26] Mack DR, Ahrne S, Hyde L, Wei S, Hollingsworth MA (2003) Extracellular MUC3 mucin secretion follows adherence of Lactobacillus strains to intestinal epithelial cells in vitro. Gut 52: 827–833. 12740338 10.1136/gut.52.6.827 PMC1773687

[27] Mack DR, Michail S, Wei S, McDougall L, Hollingsworth MA (1999) Probiotics inhibit enteropathogenic *E. coli* adherence in vitro by inducing intestinal mucin gene expression. Am J Physiol 276: G941–950. 10198338 10.1152/ajpgi.1999.276.4.G941

